# Prediction modelling in the early detection of neonatal sepsis

**DOI:** 10.1007/s12519-021-00505-1

**Published:** 2022-01-05

**Authors:** Puspita Sahu, Elstin Anbu Raj Stanly, Leslie Edward Simon Lewis, Krishnananda Prabhu, Mahadev Rao, Vijayanarayana Kunhikatta

**Affiliations:** 1 Department of Pharmacy Practice, Manipal College of Pharmaceutical Sciences, Manipal Academy of Higher Education (MAHE), Manipal, 576104 Karnataka India; 2 Department of Paediatrics, Kasturba Medical College, Manipal Academy of Higher Education (MAHE), Manipal, Karnataka India 576104; 3 Department of Biochemistry, Kasturba Medical College, Manipal Academy of Higher Education (MAHE), Manipal, Karnataka India 576104

**Keywords:** Neonatal sepsis, Predictors, Prediction modelling, Systematic review, Validation

## Abstract

**Background:**

Prediction modelling can greatly assist the health-care professionals in the management of diseases, thus sparking interest in neonatal sepsis diagnosis. The main objective of the study was to provide a complete picture of performance of prediction models for early detection of neonatal sepsis.

**Methods:**

PubMed, Scopus, CINAHL databases were searched and articles which used various prediction modelling measures for the early detection of neonatal sepsis were comprehended. Data extraction was carried out based on Critical Appraisal and Data Extraction for Systematic Reviews of Prediction Modelling Studies checklist. Extricate data consisted of objective, study design, patient characteristics, type of statistical model, predictors, outcome, sample size and location. Prediction model Risk of Bias Assessment Tool was applied to gauge the risk of bias of the articles.

**Results:**

An aggregate of ten studies were included in the review among which eight studies had applied logistic regression to build a prediction model, while the remaining two had applied artificial intelligence. Potential predictors like neonatal fever, birth weight, foetal morbidity and gender, cervicovaginitis and maternal age were identified for the early detection of neonatal sepsis. Moreover, birth weight, endotracheal intubation, thyroid hypofunction and umbilical venous catheter were promising factors for predicting late-onset sepsis; while gestational age, intrapartum temperature and antibiotics treatment were utilised as budding prognosticators for early-onset sepsis detection.

**Conclusion:**

Prediction modelling approaches were able to recognise promising maternal, neonatal and laboratory predictors in the rapid detection of early and late neonatal sepsis and thus, can be considered as a novel way for clinician decision-making towards the disease diagnosis if not used alone, in the years to come.

**Supplementary Information:**

The online version contains supplementary material available at 10.1007/s12519-021-00505-1.

## Introduction

Neonatal sepsis is the third most prominent cause of mortality among neonates after intrapartum complications and prematurity. Nearly, 13% of neonatal mortality is bequeathed by it, of which 42% of the death occurs in the first week itself [1]. The primary challenge in neonatal sepsis is its evasive signs and symptoms which makes the diagnosis and prognosis burdensome. The only unrivalled quick fix is blood culture confirmation which takes virtually two days to generate result [2]. The necessity of a distinguished biomarker is fundamental for meticulous and for the nick of time diagnosis [3]. Inadequate immunity in neonates makes it soldier to overcome mortality [4]. Therefore, there is a need to glance at novel approaches to embark upon the situation.

Prediction modelling is a statistical measure of employing established results to generate, design and validate a model that can be applied for anticipating expected outcomes [5]. It can greatly assist clinician as well as the health-care professionals on efficient management of any disease [6]. Severity score generated by these prognostic prediction models aids in scrutinising the profoundness of the disease in due time. Furthermore, it recuperates any disease management by risk and patient stratification [7].

Prediction modelling has reinforced in the pronouncement of several potential biomarkers in the diagnosis of neonatal sepsis which essentially comprehend C-Reactive Protein (CRP), Interleukin-27, neutrophil CD64, etc. [8–10]. It also inculcates myriad maternal and neonatal risk factors such as intrapartum temperature, gestational age at delivery, duration of premature rupture of membrane (PROM), intrapartum antibiotic treatments, mode of delivery, birth weight, etc. in the conjecture of the disease [11–13]. Similarly, clinical and laboratory biomarkers such as maternal white blood cells, absolute neutrophil count was also used for the diagnosis of the disease [13]. Incorporation of laboratory diagnostic markers such as WBC along with standard biomarker such as CRP in the prediction model has considerably reduced the applicability of antibiotics in early-onset neonatal sepsis [14]. Sepsis risk calculator, scoring system generation were some of the desired results of prediction modelling implementation [15, 16]. Similarly, application of sepsis risk calculator through prediction model development also resulted in decrement in the usage of antibiotic therapy [15].

Hence, a systematic review is required to assess this modernistic approach which will give a fresh insight in the prognosis of having neonatal sepsis. The principal purpose of the study is to provide an overall depiction of the entire prediction modelling measures projecting the early detection of neonatal sepsis. As per our proficiency, this would be the aboriginal systematic review to comprehend this facet. The current systematic review will benefit the young researchers and investigators to have an eye at the multifarious pathway to undertake their research in neonatal sepsis meanwhile making a provision for the clinician for improvised supervision on the disease.

## Methods

### Information sources

Articles were searched from distinct databases such as PubMed, Scopus, and CINAHL using the following keywords “Prediction model” “neonatal sepsis” “neonatal sepses”. Earliest ten-year papers were searched till September 2020 for the study purpose in all the databases.

### Eligibility criteria

Papers were comprehended if they fit the following inclusion criteria: (1) Operational definition of neonatal sepsis: Neonates having positive blood culture before 72 h of life were considered as early neonatal sepsis, whereas neonates having positive blood culture report after 72 h of life were termed as late neonatal sepsis. Meanwhile, neonates having positive blood culture report before 30 days of life were considered as neonatal sepsis patient [17]; (2) Case–control, cohort—prospective/retrospective studies predicting the prognosis of having neonatal sepsis (early/late onset) which is to be culture positive; (3) Prediction model developed through various statistical procedure like machine learning/logistic regression/artificial intelligence for early detection of neonatal sepsis; (4) The prediction developmental model should be either internally or externally validated, and (5) Articles published in English peer reviewed journal.

However, randomised clinical trial, review/systematic review/meta-analysis related to early/late neonatal sepsis were excluded from the study. Implementation of the prediction models pertaining to neonatal sepsis or prognosis of the disease was also ruled out from the study.

### Study selection

The study screening was conducted in accordance with the Preferred Reporting Items for Systematic Review and Meta-analysis (PRISMA) guidelines [18]. The first reviewer [P.S] evaluated all the search results. Further title, abstract and  subsequently full text screening were cross checked by the rest two reviewers (EAR, VK).  Title, abstract and full text screening was consummated on the ground of study design/disease/outcome/intervention/irrelevant. Any difference in the opinion regarding final included articles was resolved through discussions.

### Data extraction and quality assessment

Data extraction was carried out by all the reviewers based on Critical Appraisal and Data Extraction for Systematic Reviews of Prediction Modelling Studies (CHARMS) checklist [19, 20]. Extricate data consisted of objective, study design, patient characteristics, type of statistical model, predictors in the model, outcome measure, sample size and location. Significant predictors were identified by relative risk or odds ratio. Outcome measures were assessed by area under the curve, sensitivity, specificity, positive predictive and negative predictive value. Prediction model risk of bias assessment tool (PROBAST) was applied to gauge the risk of bias of the included articles and were classified into high, low, and unclear risk of bias. PROBAST risk of bias consists of questions from four domains which include participants, predictors, outcome and analysis. A subtotal of 20 questions were present for the judgement of the paper. These questions were answered as yes/no/probably yes/probably no/ unclear, where “yes” implies the absence of bias, while “no” signifies the presence of bias. An article is denoted as “low risk of bias”, if all the desirable answers to the 20 questions were yes or else it is designated as high risk. An unclear answer to any of the 20 questions represents the article to be of unclear risk of bias [21].

### Data synthesis and analysis

Systematic analysis of all the included studies was conducted to generate all the desired data. Statistical measure employed for building the model was determined to figure out the type of it. Predictors were viewed to identify its potential in the model. Performance of the prediction model were determined by noting the major indices like Area Under the curve (AUC), Specificity, Sensitivity, Negative Predictive Value (NPV) and Positive Predictive Value (PPV). External and internal validation of the models was also scrutinised to determine the efficiency of the model.

## Results

**Fig. 1:**
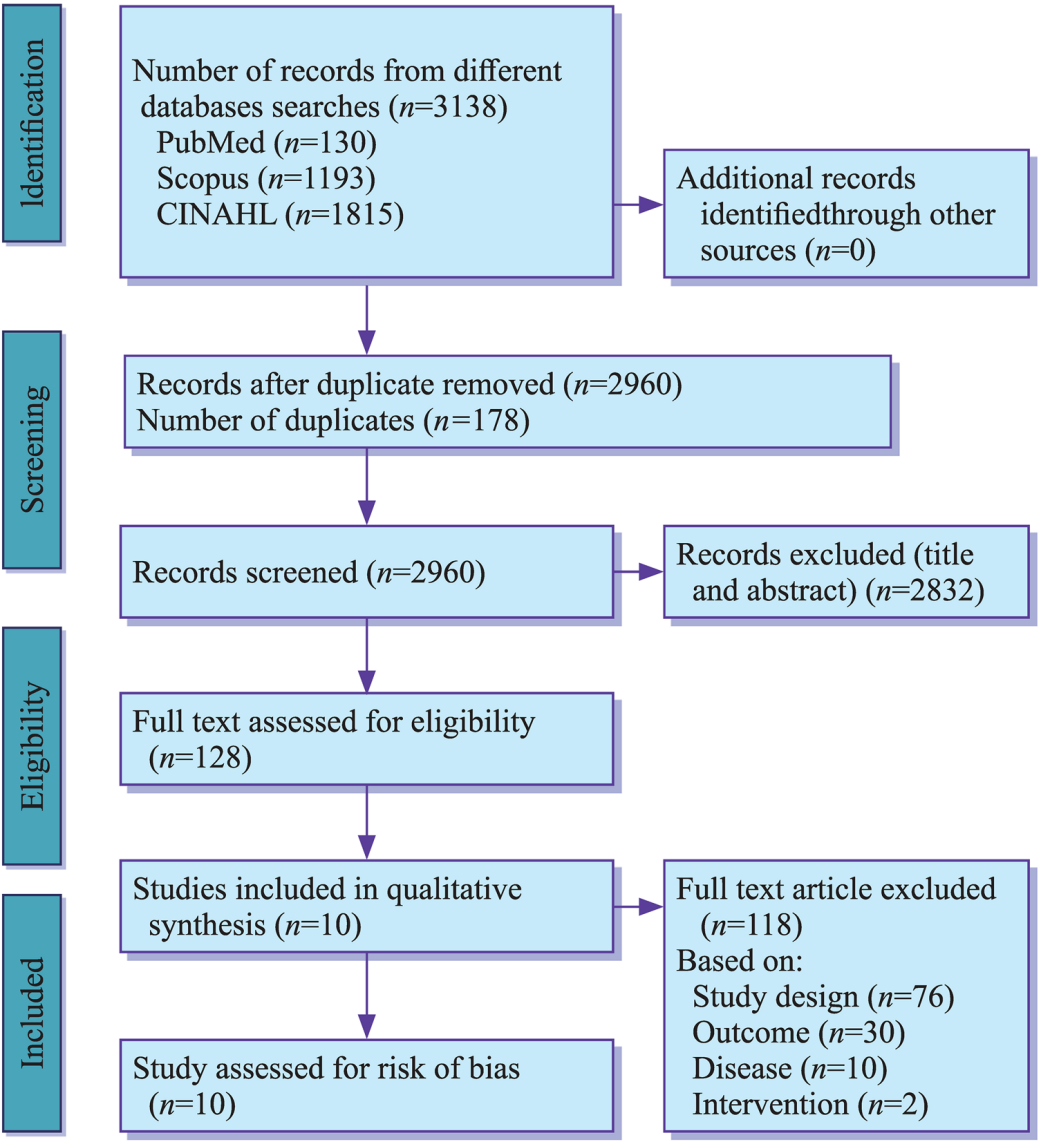
Preferred reporting item for Systematic Review and Meta Analyses flow diagram of the included studies from PubMed, CINAHL, and SCOPUS

The study screening was conducted in accordance with the Preferred Reporting Items for Systematic Review and Meta-analysis (PRISMA) guidelines. A total of 3135 articles were retrieved from three databases, i.e. PubMed, CINAHL and Scopus. An aggregate of ten studies were incorporated for systematic review after scrutinising on the basis of eligibility criteria. The screening process has been detailed in Fig. 1.

### Study characteristics

**Fig. 2:**
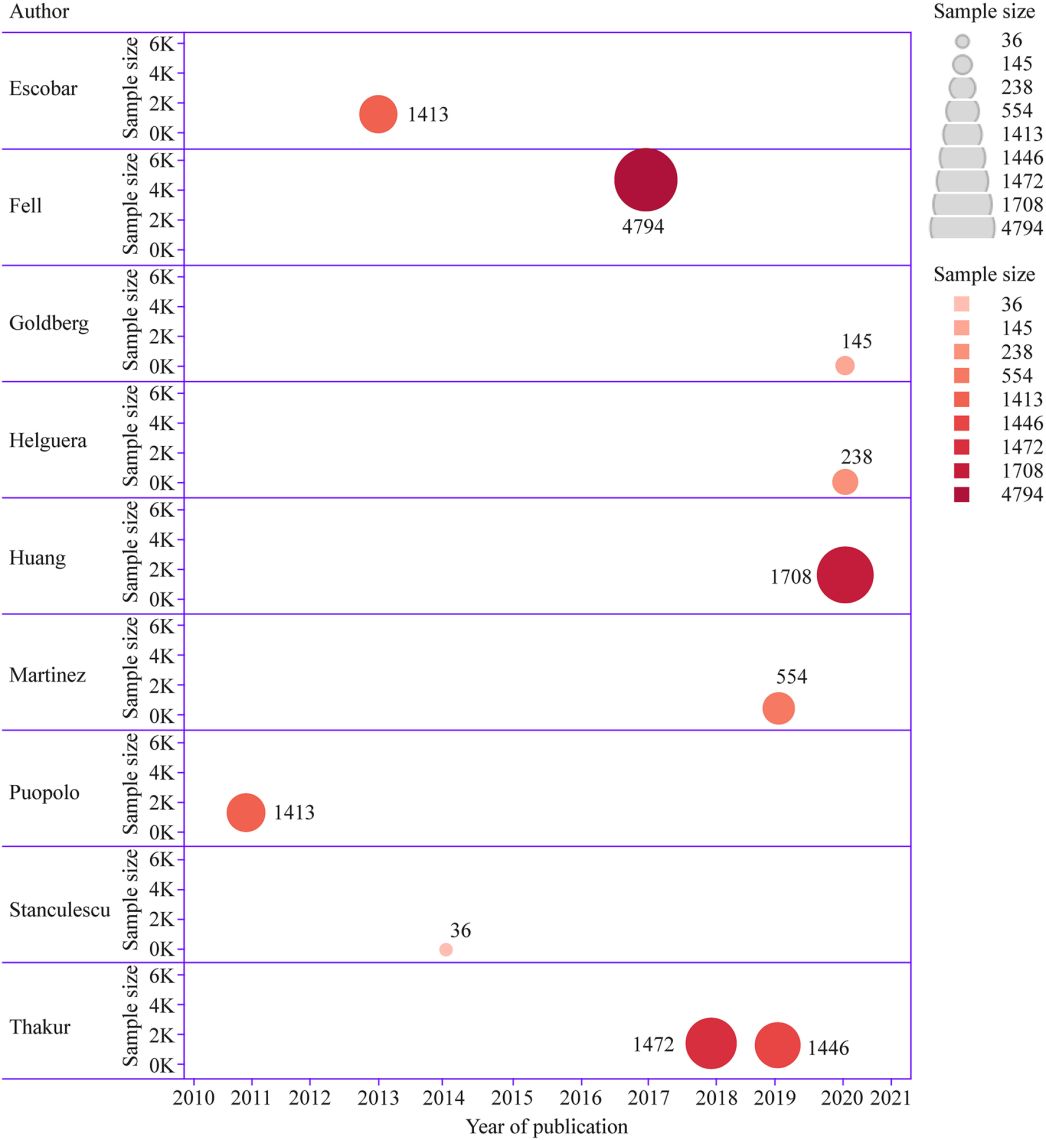
Year-wise publication of all the included articles with respect to sample size, where sample size is represented by colour gradient and size of circle

Majority (five studies) of the included articles were performed in United State of America [24, 26, 29, 30], while the remaining each were from Israel, China, Mexico, United Kingdom and Canada [22, 23, 27, 28, 31]. The highest sample size of all the included studies was 4794, while the minimal is 36, which are illustrated in Fig. 2. Most of the studies were single centre (six studies), while 30% (three studies) were of multicentre origin. Greater number of studies (seven studies) had applied logistic regression to build a prediction model, while two of the studies had applied artificial intelligence. Autoregressive hidden Markov model (AR-HMM) was applied by a single study for prediction model building [28]. Partly, the studies (five studies) had applied split validation (70:30 ratio) concepts for the derivation and validation of the model, while one of the studies had applied external validation [21]; while the other four studies had utilised bootstrap or concordance statistics methodology for internal validation.

Neonatal sepsis detection was the primary outcome of considerable number of studies (five studies) [27–31]. Early-onset and late-onset sepsis detection were encountered as the main outcome in three and two different studies, respectively [22–26]. Sheer single study had stratification of the early-onset neonatal sepsis patients based on the degree of illness as the defined outcome [25]. The length of the age of all the participants during outcome assessment varied from 0 to 30 days of life. The major characteristics of the included studies are summarised in Tables 1, 2 and 3. Predictors adopted in the comprehended studies for constructing the model were classified into four domains such as neonatal factors, maternal factors, clinical, laboratory parameters and miscellaneous. Table 4 represents the predictors notified in each study for the final model prediction.

**Table 1:** Main characteristics of the included articles for quick detection of late onset sepsis (LOS)

Study ID	Aims	Subject and study Characteristics (design, study period)	Type of prediction model	Potential predictors	Results/major findings
Goldberg et al. [22]	To use clinical and laboratory evaluation to assess sepsis risk	Prospective case control, single centre, Jan 2016–Jun 2019 Neonates with late onset sepsis confirmed by blood culture results	Multivariate logistic regression	Sick appearance, neutrophil to lymphocyte ratio > 1.5, CRP > 0.5 mg/dL, central line, abnormal physical examination, tachycardia, abnormal body temperature, hyperglycaemia, abnormal WBC and neutrophil count, lymphopenia, thrombocytopenia	Entire study cohort into 2:1 ratio 31 cases, 62 controls in the developmental cohort while 17 cases, 35 controls in the validation cohort Out of all the potential univariate predictors- 3 independent predictors associated with late onset neonatal sepsis i.e., sick appearance (OR 5.7, 95% CI 1.1–29.1), CRP > 0.75 (OR 5.4, 95% CI 1.1–26.3), neutrophil to lymphocyte ratio > 1.5 (OR 6.7, 95% CI 1.2–38.5) Derivation model AUC = 0.92, validation model AUC = 0.90
Huang et al. [23]	Development and validation of a nomogram for predicting late onset neonatal sepsis	Mixed retrospective and prospective cohort study, multicenter, Jan 2014–Dec 2017 and Jan 2018–Dec 2018 Neonates with gestation age < 37 week and is admitted to any of the three hospital within 24 h after birth	Logistic regression- backward stepwise method	Age of mother, maternal and antenatal glucocorticoid treatment, PROM, antibiotic treatment before delivery, gestational diabetes and hypertension, delivery season, method of delivery, multiple pregnancy, gestational age, birth weight, gender, asphyxia, use of dopamine, albumin, antibiotics, start day of enteral nutrition, endotracheal intubation, mechanical ventilation, PICC, UVC, thyroid hypo functions	Developmental cohort: Jan 2014 to Dec 2017; Validation cohort: Jan 2018 to Dec 2018 Development cohort (total 1256 samples:96 late onset sepsis, 1160 without late onset sepsis); Validation cohort (452 sample-34 late onset sepsis, 418 no late onset sepsis) Final model showed endotracheal intubation (RR 5.195), thyroid hypofunction (RR 4.084), UVC (RR 1.346), birth weight (RR 0.136) were associated with late onset sepsis Developmental cohort used to build the nomogram and validation cohort to test the nomogram NOMOGRAM A include thyroid function, birth weight, endotracheal intubation, UVC while NOMOGRAM B includes birth weight, UVC, endotracheal intubation The AUC value of nomogram A [development: C-index = 0.855 (0.802–0.907) sensitivity 0.500, specificity 0.918, NPV-0. 850, PPV-0.667; validation: C-index = 0.834 (0.775–0.894) sensitivity- 0.453, specificity-0.923, NPV- 0.839, PPV-0.660] is larger than that for Nomogram B [development: C-index = 0.793 (0.693–0.893); validation: C-index = 0.765 (0.660–0.870)] [development: *P* = 0.028; validation: *P* = 0.0264] Thus, NOMOGRAM A showed better prediction for late onset neonatal sepsis with thyroid function inclusion

*AUC* Area under the curve, *CI* confidence interval, *CRP* C reactive protein, *LOS* late onset sepsis, *OR* odds ratio, *PICC* peripherally inserted central catheter, *PROM* premature rupture of membrane, *RR* relative risk, *UVC* umbilical venous catheter, *WBC* white blood cell

**Table 2:** Main characteristics of the included articles for quick detection of early onset sepsis (EOS)

Study ID	Aims	Subject and Study Characteristics (design, study period)	Types of prediction model	Potential predictors	Results/major findings
Puopolo et al. [24]	Developing a quantitative model for Early onset neonatal sepsis on basis of intrapartum maternal risk factors	Nested case control, multicentre, Jan 1, 1993–Dec 31 2007 Infants born at > = 34-week gestation who had positive blood or CSF culture result for a pathogenic bacteria before 72 h of life	Multivariate logistic regression	Maternal gravidity, parity, delivery mode, GBS status, duration of rupture of membrane, maternal intrapartum temperature, presence of MSAF, maternal hypertension, maternal intrapartum medications, obstetric anaesthesia	Model development with 210 cases and 659 controls, while validation set with 140 cases and 404 controls Two most important predictors were antepartum temperature (58% of contribution) [OR 3.41 (2.23–5.20)) and gestational age (17%) [OR 1.09 (1.05–1.13) Final predictors in the model are gestational age, GBS carrier status, duration of PROM, intrapartum temperature and intrapartum antibiotic treatment c statistics = 0.80 in total (Development model c = 0.807, validation *c* = 0.794)
Escobar et al. [25]	Quantitative stratification algorithm for early onset neonatal sepsis in new born with > 34 week of gestation	Nested case control, multicentre, Jan 1, 1993–Dec 31, 2007 Infants born at ≥ 34 week of gestation who had positive blood or CSF culture result for a pathogenic bacteria before 72 h of life	Multivariate logistic regression, recursive partitioning	Maternal ethnicity, multiple gestation, gestational Age, gender, birth Weight, mode of delivery, APGAR score (< 7 at 5 min), clinical status (6, 12, 24 h) i.e., clinically ill, equivocal and well appearing	Model development with 183 cases and 569 controls while derivation set with167 cases and 494 controls Three categories used for risk stratification of sepsis risk at birth estimated from maternal factors i.e., clinical illness, equivocal and well appearing Three categories: 1) < 0.65/1000 live births, 55.7% cases; 2) 0.65–1.54/1000 live births, 23.1% cases 3) > 1.54/1000 live births, − 21.7% cases < 0.65/1000 live births (well appearing85% cases continued observation, equivocal presentation: 11% observe and evaluate clinical illness (in total: 4% treat empirically) 1) < 0.65 category A) well appearing: PP 0.11, NNT-9370, B) Equivocal presentation: PP 1.31, NNT = 763 C) Clinical illness: PP 4.66, NNT = 214 2) 0.65–1.54 category A) well appearing PP 1.08, NNT-923, B) Equivocal presentation: PP 11.07, NNT = 90 C) Clinical illness: PP 62.94, NNT = 16 3) > 1.54 category A) well appearing: PP 6.74, NNT-148, B) Equivocal presentation: PP 11.07, NNT = 90 C) Clinical illness: PP 62.94, NNT = 16
Martinez et al. [26]	To develop a neural network classification model for EONS detection	Retrospective, single centre, 2016–17 Preterm and term neonates with confirmed positive culture results within 72 h of life	Artificial neural network (deep learning) model approach statistical method- linear regression	Neonatal variable: premature, gender, weight < 1500 and 2500 g, APGAR score < 7 at 1 and 5 min, respiratory distress Obstetric variable: Gestational Age, number of prenatal controls, number of pregnancies, birth c section type of birth, IUGR background, assistant for prenatal control, assistant for at least 3or 4 prenatal control, PROM > 6 or 18 h, chorioaminocentesis, multiple pregnancy Maternal Infectious disease: maternal fever, yeast infection, UTI, STD	186 cases and 369 controls Model sensitivity: 80.32%, specificity: 90.4%, PPV:83.1% NPV: 88.7%, useful for detecting positive and true negative cases Model AUC:92.5% confirms adequacy of model Accuracy: 86.74% correctly identify positive and negative cases

*APGAR* appearance, pulse, grimace, activity and respiration, *AUC* area under the curve, *CSF* cerebrospinal fluid, *EOS*: early onset sepsis, *GBS* Group B Streptococcus, *IUGR* intrauterine growth retardation, *MSAF* meconium-stained amniotic fluid, *NNT* number needed to treat, *OR* odds ratio, *NPV* negative predictive value, *PP* posterior probability, *PPV* positive predictive value, *PROM* premature rupture of membrane, *STD* sexually transmitted disease, *UTI* urinary tract infection

**Table 3:** Main characteristics of the included articles for quick detection of neonatal sepsis

Study ID	Aims	Subject and Study Characteristics (design, study period)	Type of prediction model	Potential predictors	Results/major findings
Helgueraet al [27]	To develop early onset and late onset sepsis (EOS, LOS) diagnostic model based on maternal and neonatal records	Observational retrospective study, single centre, 2017–18 18 months) Preterm and term neonates with confirmed positive culture results	Artificial intelligence- artificial neural network approach statistical method- linear regression	Maternal: maternal age, maternal morbidity, cervicovaginitis, UTI, PROM, Chorioaminocentesis Neonatal: gestational age, Birth Weight, apnoea, fever, hypothermia, tachycardia or bradycardia, tachypnea or bradypnea, apnoea, neutrophils count, mechanical ventilation, received pharmacological treatment, platelet count, gender, band cells and band cells percentage, relation band/neutrophil, catheter use, immature/total neutrophil ratio, band neutrophil/segmented neutrophil	238 neonates (22 EOS, 84 LOS) 132 non- septic, 106 sepsis Best performance for sepsis model contains 25 maternal and neonatal factors When the value of prediction is > 0.85 then the patient is termed as septic Maternal age, neonatal fever, apnoea, platelet count-most important predictor of sepsis followed by cervicovaginitis, gender, bradypnea, band cells, catheter presence, Birth Weight, neutrophil counts, fetal morbidity Total model *R*^*2*^ = 0.974, specificity model 80%, sensitivity 93.33%, Accuracy 86.66%, precision 82.35%, NPV 92.3%, PPV 82.35%, AUC 94.44% Physician performance- specificity—46.67%, sensitivity—100, AUC—73.33%, PPV 65.22%, NPV 100%
Stanculescu et al. [28]	Whether physiological events can detect neonatal sepsis prior to blood culture	Case control, single centre, 2008–11 Very low weight birth baby admitted to NICU	Auto regressive- hidden markov model (AR-HMM)- forward backward algorithm	Bradycardia, desaturation, mini bradycardia, oximeter error, x- miscellaneous factors	Baby generated physiological events display a higher incidence in sepsis group Amount of people handling does not differ much in control and sepsis group. Same results for oximeter error and x factor Sepsis group showed increased number of brady and minibradycardia before positive culture Filtering data [AR-HMM missing data (md) AUC = 0.74] **[**AR-HMM without missing data (wmd) AUC = 0.72], Validation AR: HMM md average precision (AP) = 0.59, *F* score = 0.61 AR-HMM wmd AP = 0.56, *F* score = 0.59
Thakur et al. [29]	To develop non-invasive model for neonatal sepsis	Retrospective, single centre, 2001–12 Neonates with one positive blood or Cerebrospinal fluid (CSF) report and age at the time of blood report should be < 28 days	Forward wald stepwise logistic regression analysis	NI model parameters: blood pressure systolic (max), diastolic (max, min, mean), heart rate (mean), respiratory rate (max, min, mean), spo2 (Max, mean), gender, temperature (max, mean), birth weight O model parameters: blood pressure Systolic (max), diastolic (max, min, mean), temperature (max, mean), Respiratory Rate (max, mean), Bands (min, max), UVC, platelet (min)	Derivation sample = 1012, validation sample = 434 2 models built (NI and O model) Parameters included in final model of NI model- Heart Rate (max, min), Birth Weight, temperature (min), gender, sPO2 (min), Blood Pressure systolic (min, mean) Parameter included in O final model-Blood Pressure systolic (min, mean), temp (min), Respiratory Rate (min), bands, platelet(max), UVC NI model AUC: 0.879 and O model AUC: 0.861, validation AUC: 0.763, 0.767, respectively Training set Model O: Sensitivity = 34.35, specificity = 97.16, PPV = 64.29, NPV = 90.86, PLR = 12.09, NLR = 0.68 Model NI: Sensitivity = 38.93, specificity = 97.16, PPV = 67.11, NPV = 91.44, PLR = 13.70, NLR = 0.63 Testing Set Model O: Sensitivity = 29.17, specificity = 97.67, PPV = 60.87, NPV = 91.75, PLR = 12.54, NLR = 0.63 Model NI: Sensitivity = 33.33, specificity = 97.93, PPV = 66.67, NPV = 92.21, PLR = 16.13, NLR = 0.68
Thakur et al. [30]	To develop and compare two prognostic model to predict sepsis by non-invasive and invasive type	Retrospective, single centre, Jun 2001–Oct 2012 Neonates with age < 30 days with one positive blood or CSF report	Forward Wald stepwise logistic regression	(Temperature, heart rate, blood pressure): Non-invasive model, (Platelet, WBC counts and bands): Invasive model	Derivation sample: 1061, Validation sample 411 AUC derivation set of invasive model and non-invasive model is 0.777, 0.824, respectively AUC Validation set of invasive models and non-invasive model is 0.830, and 0.824, respectively Both models significant at *P* < 0.001 Invasive model: Derivation set: sensitivity = 32, specificity = 97.2 Validation set: sensitivity = 27.1, specificity = 96.7 Non-invasive model: Derivation set: sensitivity = 29.3, specificity = 97.9 Validation set: sensitivity = 35.4, specificity = 96.4
Fell et al. [31]	To identify cases of neonatal sepsis using new born screening	Cohort, single centre, Jan 1, 2010–Dec 31, 2015 Neonates with term birth 37 week, late preterm birth 34–36 week, early preterm < 34 week	Multivariable logistic regression	Model 1 predictor: sex, gestational age, birth weight, plurality and Total parental nutrition Model 2 predictor: Model 1 variable + foetal to adult Haemoglobin level Model 3: Model 2 variable + 17-OHP + TSH Model 4: Model 3 variables + restricted cubic spline terms for the top 5 ranked analysts/analyte ratio until maximum number of parameters were reached	3 major models: Term birth (> 37 gestational age) Model 1, 2, 3, 4 Late preterm birth (34–36 gestational age) Model 1, 2, 3, 4, Early preterm birth (< 34 gestational age) Model 1, 2, 3, 4 Model 1 (term birth): c statistic adjusted: model 1a: 0.577 AIC: 19,372, model 1b: 0.577 AIC: 19,372, model 3a: 0.704 AIC: 17,977, model 4a: 0.848 AIC: 13,788 Model 2 (Late preterm 34–36 week): c statistic model 1b: 0.683 AIC: 7722, Model 2b: 0.685 AIC: 7711, model 3b: 0.725 AIC: 7588, Model 4b: 0.782, AIC: 7086 Model 3 (early preterm birth < 34 week): c statistics model 3a: 0.650 AIC: 6696, model 3b: 0.649, AIC: 6695, model 3c: 0.654, AIC: 6678, model 4c: 0.667 AIC: 6559 Lesser the AIC, better is the result

*AIC* Akaike information criterion, *AR HMM* Auto regressive hidden Markov model, *AUC* area under the curve, *CSF* cerebrospinal fluid, *Max* maximum, *Min* minimum, *NLR* negative likelihood ratio, *NPV* negative predictive value, *OHP- 17* hydroxyprogesterone, *PLR* positive likelihood ratio, *PPV* positive predictive value, *TSH* thyroid stimulating hormone, *UVC* umbilical vein catheter, *WBC* white blood cell

**Table 4:** Summary of the potential predictors utilized in the included articles

Factors	Goldberg et al	Huang et al	Puopolo et al	Escobar et al	Martinez et al	Helguera et al	Stanculescu et al	Thakur et al	Thakur et al	Fell et al
Birth weight	–	–	√	#	#	√	–	√	√	√
Gender	–	–	–	#	#	√	–	√	–	–
Gestational age	–	#	√	#	#	#	–	–	–	√
Sick appearance	√	–	–	–	–	–	–	–	–	–
Neonatal fever	–	–	–	–	–	√	–	–	–	–
Bradycardia	–	–	–	–	–	√	√	–	–	–
Apnoea	–	–	–	–	–	√	√	–	–	–
Heart rate	–	–	–	–	–	–	–	√	√	–
Blood Pressure	–	–	–	–	–	–	–	√	√	–
Temperature	#	–	–	–	–	–	–	√	√	–
Haemoglobin	–	–	–	–	–	–	–	–	–	√
CRP	√	–	–	–	–	–	–	–	–	–
Neutrophil count	#	–	–	–	–	√	–	–	–	–
N/L ratio	√	–	–	–	–	–	–	–	–	–
Platelet count	–	–	–	–	–	√	–	√	–	–
Thyroid function	–	√	–	–	–	–	–	–	–	√
Band cells	–	–	–	–	–	√	–	–	√	–
Maternal age	–	–	–	–	–	√	–	–	–	–
Intrapartum temperature	–	–	√	–	–	–	–	–	–	–
Antepartum temperature	–	–	√	–	–	–	–	–	–	–
GBS status	–	–	√	–	–	–	–	–	–	–
PROM duration	–	#	√	–	#	#	–	–	–	–
Cervicovaginitis	–	–	–	–	–	√	–	–	–	–
Catheter	–	√	–	–	–	√	#	–	–	–
Endotracheal intubation	–	√	–	–	–	–	–	–	–	–

Predictors utilized in the included studies, where (√) signifies presence of the predictor in the final model of particular study whereas (–) signifies not applicable to the particular study, (#) signifies predictor did not show significant result in final model

*CRP* C Reactive Protein, *GBS* Group B Streptococcus, *N/L* neutrophil/lymphocyte, *PROM* Premature rupture of membrane

### Risk of bias assessment

Prediction model risk of bias assessment tool (PROBAST) was applied to appraise the risk of bias of the included articles. Six articles were of low risk of bias while two were of high due to their minimal sample size. Remaining two articles were of unclear risk of bias as “analysis domain” of the PROBAST table was unable to give a clear conclusion. Since, one of the domains was unclear, we labelled the paper as the same as per the PROBAST guidelines [21]. The detailed assessment of risk of bias for the included articles using PROBAST tool, based on individual domain is provided in the supplementary table 1.

### Predictors’ assessment

Predictors’ assessment of the included articles was evaluated on the basis of neonatal factors, maternal factors, clinical/laboratory parameters with sign and symptoms and miscellaneous factor for quick detection of early, late or neonatal sepsis as a whole.

Maternal factors like maternal age, cervicovaginitis were the potential predictors in detection of sepsis as whole [27]; whereas, group B streptococcus status, duration of premature rupture of membrane aids in evaluating early-onset sepsis [24].

Sepsis detection can be eased out by neonatal predictors like fever, birth weight, foetal morbidity and gender [27]. Birth weight, endotracheal intubation, thyroid hypofunction, umbilical venous catheter (UVC) was the promising neonatal factors for predicting late-onset sepsis [23]; while gestational age, intrapartum temperature and antibiotics treatment were utilised as budding prognosticator for early-onset sepsis detection [24].

Clinical and laboratory parameters suchlike sick appearance, neutrophil–lymphocyte ratio > 1.5, and C-Reactive Protein > 0.75 were able to detect late-onset sepsis at the earliest [22]. Signs and symptoms such as increase in the number of bradycardia and mini-bradycardia events was a reflection on fast onset of neonatal sepsis [28]. Moreover, platelet count, bradypnea, band cells heart rate (maximum, minimum), SpO_2_ minimum, blood pressure systolic (minimum, mean), thyroid, acyl carnitine, amino acids and adrenal functions were few of the indicators which also showed significant results in the early detection of neonatal sepsis as whole [27–30].

Miscellaneous factors like oximeter error did not show any significant result [28]; while, catheter presence showed positive sign in fast detection of neonatal sepsis [25].

### Outcome measures

The AUC (derivation model AUC = 0.92, validation model AUC = 0.90) noted for the model developed through clinical and laboratory parameters for the prognosis of having late-onset neonatal sepsis was quite satisfactory [22]. Formation and testing of various nomogram from the derivation and validation set (derivation AUC = 0.855, validation AUC = 0.834) for prediction of late-onset neonatal sepsis was also successful in doing so [23].

Intrapartum maternal risk factors were utilised to form a prediction model for early-onset sepsis detection with AUC of 0.807 and 0.794 from derivation and validation set, respectively [24]. Early onset sepsis identification by neural network model had AUC 92.5, accuracy of 86.74, sensitivity 80.32, and specificity of 90.4 [26]. Quantitative stratification algorithm for early-onset sepsis detection had classified neonates with sepsis risk at birth into three categories, i.e. clinical illness, equivocal and well appearing [25].

Autoregressive hidden Markov model with AUC of 0.74 clearly exhibited that physiological change in events such as bradycardia or mini-bradycardia can detect neonatal sepsis prior to culture report [26]. Maternal and neonatal factor model had predicted the neonatal sepsis disease with an AUC of 97.4, specificity 80%, sensitivity 93.33%, PPV 82.35 and NPV of 92.3 which had surpassed the performance of physician also [27]. Non-invasive model developed for early detection of neonatal sepsis through logistic regression showed better results when compared to that of the invasive model (AUC of invasive model 0.777, AUC of non-invasive model 0.824) [30]. Neonatal sepsis detection by another team of authors also displayed similar results (AUC 0.879) [29]. Newborn screening results like thyroid and adrenal function, acyl carnitine and amino acids also improved the model fit for detection of neonatal sepsis [31].

### Validation measures

Half of the included studies had utilized 2:1 ratio, i.e. split method of derivation and validation set for evaluating the accuracy, precision and validity of model [22, 24, 25, 29, 30]; meanwhile, mere one study had used the external validation procedure of conducting the same study in different set of samples at different period of time [23]. 70:15:15 (70 derivation set, 15 validations set, 15 testing set) ratio was applied for validation of model developed by maternal and neonatal risk factor for identification of neonates with sepsis. Back propagation and neural network methods were applied to do so. The performance of the model was calculated on three metrics, i.e. root mean square error (RMSE), regression coefficient and statistical slope and intercept [27]. Similar back propagation method was applied by another study for validation of model for early-onset sepsis detection [26]. Average precision and *F* score was generated by precision recurve call for validation purpose in model built by autoregressive hidden Markov model [28]. Bootstrap method of internal validation (200 samples) was applied to validate model which predicted neonatal sepsis through the analysts of newborn screening [31].

## Discussion

Failure to quickly diagnose neonatal sepsis, primarily due to its indefinite sign and symptom makes the disease more lethal and destructive. Blood culture report being the only chief solution, virtually takes two days to generate result. Therefore, there is a need to look into novel approaches, which may help in fast prediction of neonatal sepsis. This systematic review tried to capture the modernistic prediction modelling methods implied for quick detection of late/early/neonatal sepsis as whole, thus endowing a fresh insight to the approach for disease management. Thus, the main purpose of our study was to perform a systematic review which would give a detailed illustration of prediction modelling measure in the prognosis of having neonatal sepsis. To the best of our knowledge, none of the available studies had focussed on the prediction modelling methods in neonatal sepsis. To fixate our study in prediction modelling in prognosis of having neonatal sepsis, the current review illustrations were based on the foundation of three divisions, i.e. principal findings, predictors assessed, comparison among included articles based on statistical approach implied to build the model.

### Principal findings

This systematic review illustrated that few articles were available on prediction modelling for fast detection of early/late/neonatal sepsis as whole. Out of those, most articles included had displayed satisfactory results in terms of prediction of the disease. Maternal, neonatal, clinical and laboratory predictors identified through these models will assist health-care professionals in the rational management of the disease [22–24, 27, 29, 30]. Model performance for quick detection of sepsis was almost equivalent to that of physician performance [27]. Stratification of early-onset sepsis into clinically ill, equivocal and well appearing using prediction modelling had helped in modifying the treatment approach for individual categories [25]. Physiological events like increased number of bradycardia or mini-bradycardia were identified as good predictors to detect the disease prior to the culture report [28]. Thus, it can be concluded that prediction modelling measures can be used as an additional approach to clinician decision, if not used alone.

### Predictors assessed compared with other studies

Included articles in our study had either utilised clinical/laboratory parameters, neonatal/maternal risk factors, or physiological events of the neonates for building the prediction model [22–31]. Birth weight characteristics, gestational age features, role of setting, sampling strategy and concomitant interventions were some of the primary predictors assessed for late-onset neonatal sepsis by routine neonatal screening [34]. Analogous results were found in one of the included articles where birth weight, endotracheal intubation, thyroid hypofunction and umbilical venous catheter (UVC) were the promising neonatal factors for predicting late-onset sepsis [23]. Study conducted by Seidel et al. for predicting late-onset sepsis by body surface screening had pooled sensitivity of 41% (95% CI 17–70%) and specificity of 56% (95% CI 34–76%), whereas another included study in our review for predicting late-onset sepsis by clinical and laboratory evaluation had shown sick appearance (OR 5.7, 95% CI 1.1–29.1), CRP > 0.75 (OR 5.4, 95% CI 1.1–26.3)), neutrophil to lymphocyte ratio > 1.5 (OR 6.7, 95% CI 1.2–38.5) as the chief predictors with model AUC of 0.92 [22]^.^

Another systematic review was designed to evaluate the outcome of early neonatal sepsis by sepsis calculator, and had shown reduced antibiotic usage and fewer surges in mortality and readmission to hospital [32, 33]; whereas, one of the included articles in the review had different objective and had shown gestational age, intrapartum temperature and antibiotics treatment as budding prognosticator for early-onset sepsis detection [24]. Yet another included article had classified early-onset sepsis patients into various categories based on the degree of illness. Stratification of those patients had enabled the health-care professionals to render distinct treatment to various categories, thus ensuring proper management [25].

Study conducted by Liang et al. illustrated that prematurity, low birth weight and young age at presentation were some of the major factors which were associated with mortality in neonates with sepsis [35]. Artificial neural network approach was employed by another included article for analysing various maternal and neonatal predictors for early detection of neonatal sepsis and had suggested that maternal age, neonatal fever, apnoea, platelet count was the most valuable predictor of sepsis followed by cervicovaginitis, gender, bradypnea, band cells, catheter presence, birth weight, neutrophil counts and foetal morbidity.

Apart from the previously mentioned predictors, novel diagnostic marker such as neutrophil CD64, platelet to lymphocyte and neutrophil to lymphocyte can also be added to the prediction model for increasing the accuracy of diagnosis of neonatal sepsis as neutrophil CD64 when combined with standard biomarkers like CRP and WBC increases the sensitivity and accuracy of diagnosis [10, 37]. Likewise, inclusion of platelet to lymphocyte and neutrophil to lymphocyte ratio as predictors in the model will increment the diagnosing capacity of early-onset neonatal sepsis in particular [37]. Thus, addition of novel biomarker along with the standard biomarkers in the model will potentiate the predicting ability of the model.

### Comparison among included articles based on statistical approach employed to build the model

Among the two of the included articles used for the quick detection of the late-onset neonatal sepsis, Huang et al. had the most appropriate methodology which can be used in other clinical settings for building prediction model [22, 23]. Logistic regression adopted for building the model had several advantages in comparison to the other approaches as it provide probability prediction of each predictor unlike only classifying it into different levels [38]. Models generated by this approach are less likely to be over fit even with the smaller datasets. Unlike other approaches like support vector machines and decision tree, it can easily accommodate larger data set and can refurbish easily to reflect new data set [39]. External validation approach was employed by Huang et al. to measure the accuracy of the model. It ensures reproducibility and transportability of the model in other clinical settings [40, 41]. Being a multicenter origin study, it provides the build model with satisfactory power to detect late-onset sepsis in heterogeneous subjects [42]. Validation cohort AUC of 0.963 of the study also highlighted similar results [23].

Out of all the included articles for fast detection of early-onset neonatal sepsis, Escobar et al.’s study can be considered to have remarkably relevant methodology [24–26]. Similar kind of methodology was adopted by Puopolo et al. and Martinez et al. [22, 23]; however, Escobar et al.’s study can be primarily considered as it had stratified early-onset sepsis neonates into clinical illness, equivocal and well appearing. Stratification method adopted in the study will assist the clinician in providing appropriate treatment modalities and strategy for improved patient care in various subgroups [43]. Logistic regression, multicenter origin, and internal validation employed by this study had made it a desirable prediction model; however, external validation was necessary for replicating the methodology in other clinical settings.

Among the five selected articles for early detection of neonatal sepsis, three had used the same statistical measure, i.e. logistic regression [29–31], while the remaining one had used autoregressive hidden Markov model approach [28]. Autoregressive Markov model is better than simple Markov model as it identifies a greater number of sequences. It also allows various sequences to be modelled rather than only inserting or deleting it. Unlike simple Markov models, these are quite expensive and require larger seed sequences to be trained thus, is difficult to replicate [44]. Study conducted by Thakur et al. 2019, 2018 was quite similar based on sample size, validation measure and outcome viewed. However, replicating it in other settings is still questionable as external validation was not performed [29, 30]. Bootstrapping method applied by Fell et al. is one of the efficient ways of internal validation when compared to that of cross or split validation as it utilises the entire data set for validating which produces results of low variance [45] still, cannot surpass the advantages of external validation. Thus, external validation being one of the inevitable parts of prediction modelling should be taken into consideration in the future research for the applicability quotient.

### Strengths

Articles containing model validation part (internal/external) were particularly included in the review, as models without validation were of low robustness and accuracy and difficult to replicate in other settings [46–48].

### Limitations

Studies conducted in English language were only considered for the review due to which a number of articles had to be excluded and thus, can be considered as one of the shortcomings. Meanwhile, scarcity of data also affected the outcome of the review. Further research (both interventional and observational studies) is required in this field to generate more evidence.

Thus, the chief findings of this review are that prediction modelling can be considered as a novel way of approach towards the disease, which not only will help the clinician in making rationale and critical decision but will help the young researchers in considering prediction modelling as an important tool for investigation.

## Conclusions

Models generated through various statistical approaches were successful in predicting the disease prior to culture report and had attained comparable outcome to that of the physician. Stratification of patient pertaining to severity of illness for varied treatment approach is another key point of the prediction model. Outcome obtained from the various prediction models were successful in generating potential predictors that can benefit the clinician in appropriate management of the disease. Thus, it can be effectively concluded that prediction model developed through proper statistical measures can be recognised as a novel way of approaching towards neonatal sepsis detection in the years to come. However, extensive research is in need to assert the prediction modelling as a sole measure for disease management based on external validation as an aspect of reference and reproducibility.

## Supplementary Information

Below is the link to the electronic supplementary material.

Supplementary file1 (DOCX 14 KB)

## Data Availability

Data sharing is not applicable to this study as no data have been generated or analysed during the present study.
