# Supplementary material for: Prediction modelling in the early detection of neonatal sepsis
Source: World J Pediatr. 2022 Jan 5;18(3):160–75. doi: 10.1007/s12519-021-00505-1 (PMC8898244; doi:10.1007/s12519-021-00505-1)
Supplement: Supplementary file 1 — Supplementary file1 (DOCX 14 KB) [file 12519_2021_505_MOESM1_ESM.docx]

**Table 1.** Risk of bias assessment using PROBAST tool for the included articles

| **Study ID** | **Participants** | **Predictors** | **Outcome** | **Analysis** | **Risk of bias** |
| --- | --- | --- | --- | --- | --- |
| Goldberg et al | + | + | + | - | High |
| Huang et al | + | + | + | + | Low |
| Puopolo et al | + | - | + | - | Low |
| Escobar et al | + | + | + | + | Low |
| Martinez et al | + | + | + | ? | Unclear |
| Helguera et al | + | + | + | - | High |
| Stanculescu et al | ? | - | + | ? | Unclear |
| Thakur et al | + | + | + | + | Low |
| Thakur et al | + | + | + | + | Low |
| Fell et al | + | + | + | + | Low |

(+) is low risk of bias, (-) is high risk of bias, (?) is unclear risk of bias
